# Effects of Light Intensity on Root Development in a D-Root Growth System

**DOI:** 10.3389/fpls.2021.778382

**Published:** 2021-12-15

**Authors:** Yohanna Evelyn Miotto, Cibele Tesser da Costa, Remko Offringa, Jürgen Kleine-Vehn, Felipe dos Santos Maraschin

**Affiliations:** ^1^Programa de Pós-Graduação em Genética e Biologia Molecular, Universidade Federal do Rio Grande do Sul, Porto Alegre, Brazil; ^2^Departamento de Botânica, Universidade Federal do Rio Grande do Sul, Porto Alegre, Brazil; ^3^Plant Developmental Genetics, Institute of Biology Leiden, Leiden University, Leiden, Netherlands; ^4^Department of Applied Genetics and Cell Biology, University of Natural Resources and Life Sciences (BOKU), Vienna, Austria; ^5^Department of Molecular Plant Physiology, Institute of Biology, University of Freiburg, Freiburg, Germany; ^6^Center for Integrative Biological Signaling Studies, University of Freiburg, Freiburg, Germany

**Keywords:** photomorphogenesis, lateral root, sugar signaling, plant development, Arabidopsis

## Abstract

Plant development is highly affected by light quality, direction, and intensity. Under natural growth conditions, shoots are directly exposed to light whereas roots develop underground shielded from direct illumination. The photomorphogenic development strongly represses shoot elongation whereas promotes root growth. Over the years, several studies helped the elucidation of signaling elements that coordinate light perception and underlying developmental outputs. Light exposure of the shoots has diverse effects on main root growth and lateral root (LR) formation. In this study, we evaluated the phenotypic root responses of wild-type Arabidopsis plants, as well as several mutants, grown in a D-Root system. We observed that sucrose and light act synergistically to promote root growth and that sucrose alone cannot overcome the light requirement for root growth. We also have shown that roots respond to the light intensity applied to the shoot by changes in primary and LR development. Loss-of-function mutants for several root light-response genes display varying phenotypes according to the light intensity to which shoots are exposed. Low light intensity strongly impaired LR development for most genotypes. Only *vid-27* and *pils4* mutants showed higher LR density at 40 μmol m^–2^ s^–1^ than at 80 μmol m^–2^ s^–1^ whereas *yuc3* and *shy2-2* presented no LR development in any light condition, reinforcing the importance of auxin signaling in light-dependent root development. Our results support the use of D-Root systems to avoid the effects of direct root illumination that might lead to artifacts and unnatural phenotypic outputs.

## Introduction

Root development takes place underground in the darkness. However, the root architecture of photomorphogenic seedlings suggests that root morphology is modulated by long-distance signals derived from aboveground tissues. Plant hormones, photosynthates, and, more recently, proteins have been directly linked to the long-distance signaling between shoots and roots (Lee et al., 2017; van Gelderen et al., 2017). Photosynthetic sugars are essential shoot long-distance signals that promote root growth (Kircher and Schopfer, 2012), and sucrose alone can promote primary root growth in the dark. Nevertheless, sucrose alone cannot fully restore the root photomorphogenic growth. The majority of studies with Arabidopsis seedlings utilize Petri dishes that allow the growth of plants under controlled conditions. Unfortunately, the full illumination of the plant, such as the roots, does not mimic the natural condition. Direct root illumination generates a burst of reactive oxygen species (ROS) (Yokawa et al., 2011), alters cytokinin homeostasis (Novák et al., 2015), and can lead to artifacts that mask relevant phenotypes toward nutrient starvation as well as responses to salt and various hormones (Yokawa et al., 2014; Mo et al., 2015; Silva-Navas et al., 2015; Wan et al., 2019; Zheng et al., 2019). Regarding photomorphogenesis, plant seedlings grown in darkness show less root development compared to light-grown seedlings. Light induces auxin synthesis in young leaves, which is polarly transported to the roots where it promotes primary and lateral root (LR) development (Bhalerao et al., 2002; Salisbury et al., 2007; Swarup et al., 2008). Shoot-derived auxin activates the root meristem and establishes PIN-derived auxin gradients in the root tip acting as a key long-distance signal in light-regulated development (Laxmi et al., 2008; Vernoux et al., 2012).

On shoot illumination, photoreceptor signaling promotes the accumulation of the transcription factor ELONGATED HYPOCOTYL5 (HY5) that moves from the shoot to the roots *via* the phloem, where it induces its own expression. The positive feedback loop created by HY5 promotes root development and nitrate uptake in response to shoot illumination. In contrast, the lack of a functional HY5 protein suppresses the main root elongation as well as shoot and root biomass accumulation (Chen et al., 2016). Light intensity has a dose-dependent effect on the photomorphogenic responses of the seedling (Osterlund et al., 2000). Roots exposed to various light intensities (38–150 μmol m^–2^ s^–1^) showed a large increase in lateral and adventitious roots, together with changes in the expression of genes related to catalytic activity, hormone and light signaling, and clock-regulated pathways (Kumari et al., 2019). Phosphate starvation responses have been identified as being dependent on shoot blue-light activated cryptochromes (Gao et al., 2021), and the degree of the phenotypic response is quantitatively dependent on light intensity. It has been reported that plants can adjust root architecture using long-distance signaling pathways in response to the quality and intensity of the light stimulus (Chen et al., 2016; van Gelderen et al., 2018) as increasing light intensity was shown to promote root growth, NO_3_ uptake, and biomass accumulation (Nagel et al., 2006; Chen et al., 2016; Kumari et al., 2019).

To evaluate the effect of sucrose on root development under dark and illuminated conditions, we identified that shoot light is essential for the root effect of exogenous sugar. We also identified that varying the light intensity experienced by the shoot largely alters root phenotypes. Furthermore, we evaluated putative regulatory components controlling light-promoted root growth by revisiting the RNA-seq data from the study by Miotto et al. (2019), from where we selected candidate genes possibly involved in the dark-grown root. The root phenotypes of T-DNA insertion lines showed that light intensity affects root photomorphogenesis in a dose-dependent manner which might mask the phenotyping of relevant mutations controlling the process.

## Materials and Methods

### Plant Material and Growth Conditions

Arabidopsis Columbia (Col-0) was used as wild-type (WT), and the mutants *aiamt1* (SALK_072125C), *btb-poz* (SALK_021843C), *fkbp* (SALK_047305C), *iaa17* (SALK_065697C), *kai2* (SALK_ 128254C), *lbd29-1* (SALK_071133C), *makr6* (SALK_082476C), *nph3* (SALK_070901C), *npy4* (SALK_046452C), *nrt2.1-2* (SALK_035429C), *pils4* (SALK_065131), *pin5-5* (SM_3_28638), *pin6* (SM_3_15050), *pp2a-b* (SALK_027044), *ric1-1* (SAIL_210_E12), *roc1* (SALK_121820C), *roc5* (GK-177D02.03), *rop9* (SALK_019272C), *gef4* (SAIL_184_C08), *scar1-t1* (SALK_017554C), *cobl9-1* (SALK_099933C), *vid27* (SALK_070099), *yuc3* (SALK_030785), *yuc8* (SALK_096110), and *yuc9* (SALK_066251) are in Col-0 ecotype background and were obtained from The European Arabidopsis Stock Center (NASC)^1^. Genotyping on T-DNA insertions was performed following the SALK^2^ instructions. The primers used for genotyping can be found in Supplementary Table 1. All experiments were performed using homozygous lines for the T-DNA insertion. Seeds were sterilized, germinated, and grown as described in the study by Miotto et al. (2019) in Murashige and Skoog (Sigma–Aldrich, M5519) medium supplemented with 1.5% (w/v) agar (Merck Millipore, 107881) and 0.05% (w/v) MES hydrate (Sigma–Aldrich, M8250), pH 5.7, on vertically oriented 12 cm square plates. When indicated, seedlings were grown in complete darkness by covering the plates in aluminum foil [dark condition (D)], fully exposed to top light illumination [light condition (L)], or with their roots protected from top illumination [light–dark condition (LD)] in a modified D-Root system (Silva-Navas et al., 2015), which consisted of a black paper surrounding 3/4 of the Petri dish length as previously described (Miotto et al., 2019). Seeds were plated at the limit of the black cover so that the roots would grow into the darkened side of the plate. The light gradient reaching the increasing depths of the black cover was quantified with an LI-COR^TM^ LI-250A Portable Light Meter (Supplementary Figure 1). Plates were kept vertically and grown under different white light intensities (21 ± 3°C, 16-h photoperiod) in the range of 40–105 μmol m^–2^ s^–1^. Primary root length was measured with ImageJ (Fiji) and plotted into graphs in GraphPad Prism 9 (GraphPad Software, Inc., CA, United States).

### GUS Staining and Microscopy Analysis

Seedlings were fixed in 80% acetone for 20 min at −20°C, washed three times in water, and incubated overnight in GUS staining buffer [10 mM ethylenediaminetetraacetic acid (EDTA), 100 mM sodium phosphate (pH 7.0), 0.1% (v/v) Triton X-100, 1 mM K_3_Fe(CN)_6_, 1 mM K_4_Fe(CN)_6_, 1 mg/ml 5-bromo-4-chloro-3-indolyl-D-glucuronide] at 37°C. Subsequently, the samples were washed in water once and cleared in 70% (v/v) ethanol at room temperature before imaging with ZEISS Axio Vert microscope.

### Confocal Imaging and Quantification

Imaging was performed by using a Leica TCS SP5 confocal microscope, equipped with a hybrid detector (HyD) in addition to the standard photomultiplier tubes (PMTs). Prior to imaging, seedlings were stained with propidium iodide (0.02 mg/ml). Meristem size was defined as the distance between the quiescent center and the uppermost first rectangle cortical cell that is two times as long as it is wide.

### 1-*N*-Naphthylphthalamic Acid and Indole-3-Acetic Acid Treatments

Treatments with 1-*N*-naphthylphthalamic acid (NPA) and indole-3-acetic acid (IAA) were performed by transferring 6-day-old seedlings previously grown in a control MS medium to supplemented media. Seedlings were grown in LD and 80 μmol m^–2^ s^–1^. Primary root length was measured 8 days after transfer.

### RNA Isolation and Real-Time Quantitative PCR

RNA extraction was performed with 4- and 7-day-old seedlings. Sample harvesting was approximately performed at zeitgeber time (ZT) = 7. RNA extraction and real-time quantitative PCR (RT-qPCR) were performed as described previously (Miotto et al., 2019). All RT-qPCR values represent three biological replicates, each containing at least two technical replicates. Primer sequences that are used can be found in Supplementary Table 2.

### Statistical Analysis

**FIGURE 1:**
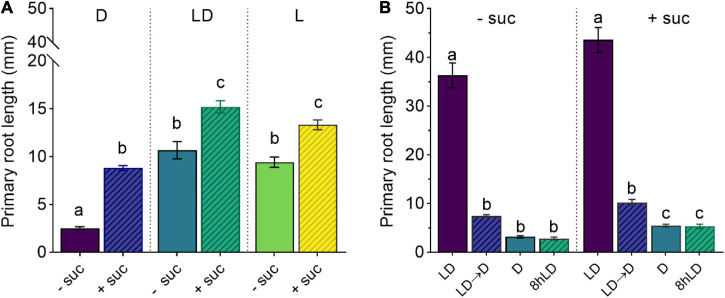
Shoot illumination is essential for sucrose-promoted primary root growth. **(A)** Primary root length of 7 days after germination (DAG) Col-0 seedlings grown with (+suc) or without (–suc) 1% sucrose in dark (D), light–dark (LD), or light (L) conditions (*n* ≥ 19). **(B)** Primary root length of 10 DAG seedlings grown with (+suc) or without (–suc) for 4 days in LD and shifted to total darkness (LD → D) or kept continuously in LD for additional 6 days (LD); alternatively, seedlings were grown for 4 days in darkness and kept in darkness for additional 6 days (D) or their shoots were briefly exposed to light for 8 h (8-h LD) (*n* ≥ 10). The letters indicate statistical significance (one-way ANOVA followed by *post hoc* Tukey’s honestly significant difference (HSD), *p* ≤ 0.05). Error bars indicate SE.

**FIGURE 2:**
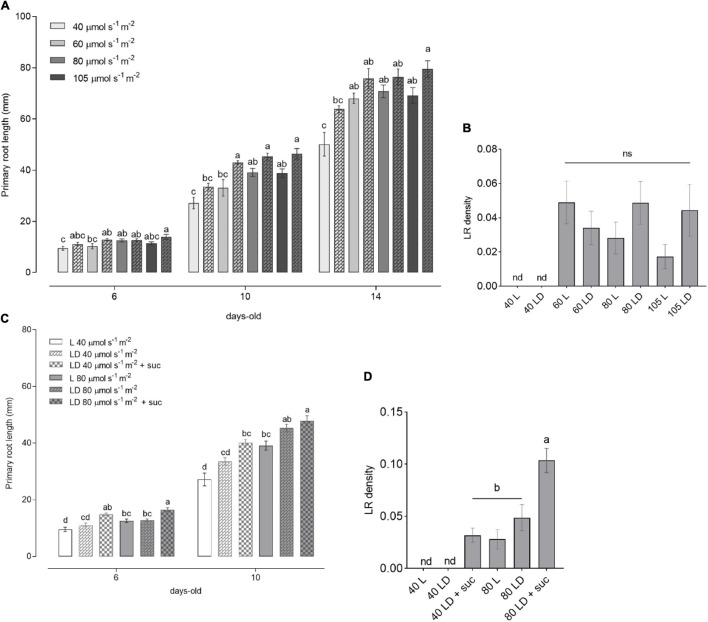
Light intensity-dependent root growth in wild-type (WT) seedlings. **(A)** Primary root length of WT seedlings grown for 6, 10, and 14 DAG (*n* ≥ 10) under increasing light intensities of 40, 60, 80, and 105 μmol m^– 2^ s^– 1^ in L (solid bars) and LD (checkered bars). **(B)** Lateral root (LR) density of seedlings from panel **(A)**. **(C)** Primary root length of WT seedlings grown in L (solid bars) and LD (checkered bars) without sucrose or with 1% sucrose supplementation in the media. **(D)** LR density of seedlings from C at 10 DAG. In panels **(A,C)**, the letters indicate statistical significance within the same time point (one-way ANOVA followed by *post hoc* Tukey’s honestly significant difference (HSD), *p* ≤ 0.05). In panels **(B,D)**, the letters indicate statistical significance within all the samples (Kruskal–Wallis with Dunn’s *post hoc* test).

**FIGURE 3:**
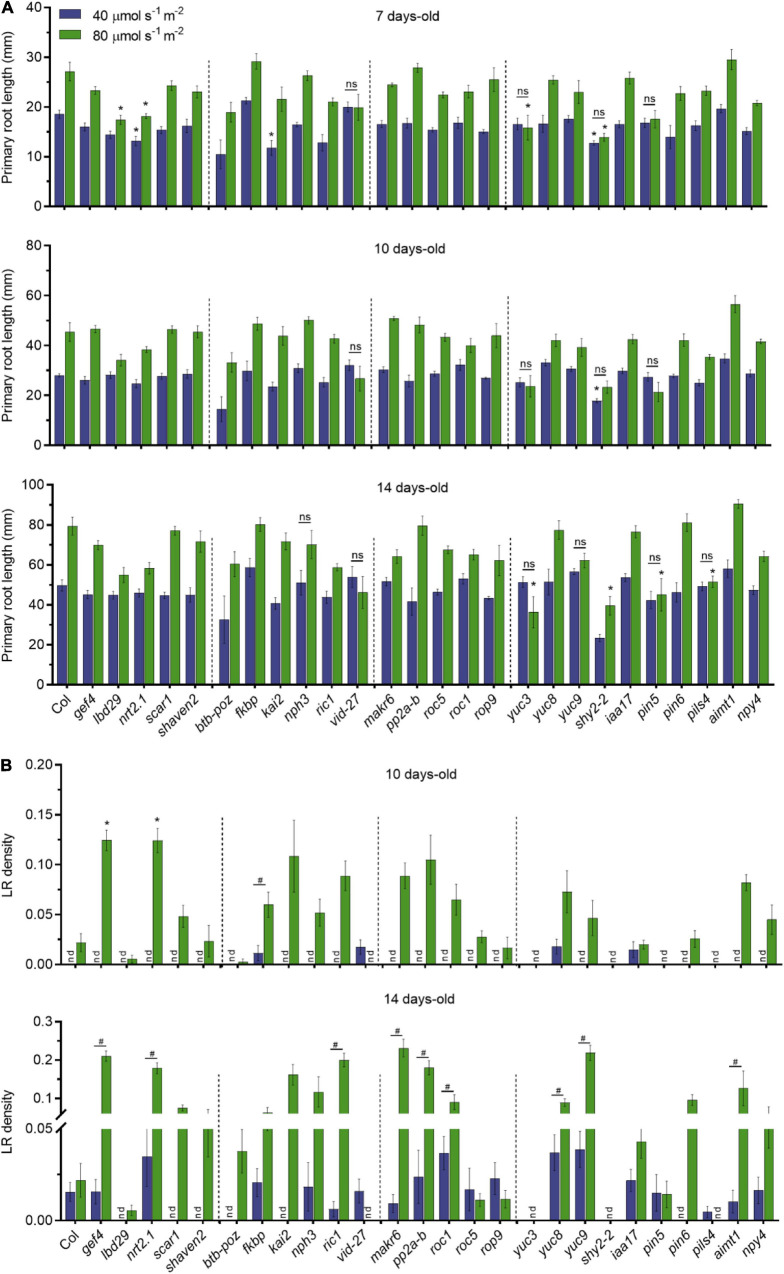
Dark-grown root of candidate genes under different light intensities in the shoots. **(A)** Primary root length of single T-DNA insertion lines (*n* ≥ 10) grown in LD conditions. Root measurements were performed at 7, 10, and 14 DAG. Error bars indicate SE. Statistical significance across genotypes is indicated with asterisks (Kruskal–Wallis with Dunn’s *post hoc* test). Non-significant statistical difference between 40 and 80 μmol m^– 2^ s^– 1^ is indicated with ns (Mann–Whitney *U* test). **(B)** LR density of seedlings from panel **(A)**; nd, not detected. Statistical significance across genotypes is indicated with asterisks (Kruskal–Wallis with Dunn’s *post hoc* test). Statistical significance between 40 and 80 μmol m^– 2^ s^– 1^ is indicated with a hashtag (Mann–Whitney *U* test, *p* < 0.05).

**FIGURE 4:**
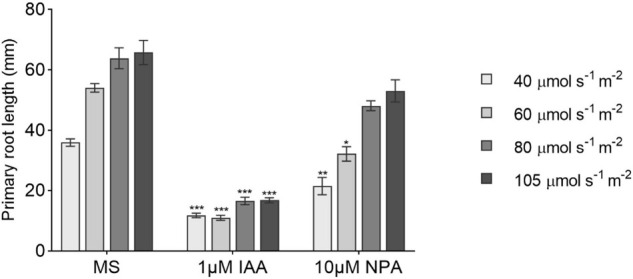
The effect of 1-*N*-naphthylphthalamic acid (NPA) and indole-3-acetic acid (IAA) on primary root growth under increasing light intensities. Primary root length at 14 DAG of seedlings grown in 1/2 MS medium (MS) or transferred to medium containing 10 μM NPA or 1 μM IAA after day 6 (*n* ≥ 8). All treatments are in LD conditions. Bars represent averages and SE. Statistical significance is indicated with asterisks within the same L condition considering MS as control (one-way ANOVA followed by *post hoc* Tukey’s honestly significant difference (HSD), *p* ≤ 0.05).

Statistical analysis was performed using GraphPad Prism 9 (GraphPad Software, Inc., CA, United States). Data were tested for normal distribution by Shapiro-Wilk test and then applied the respectively statistic test, when significant (*p* ≤ 0.05) were showed in the graphs. The statistical details of each experiment (i.e., the test used and replicates) can be found in section “Results” and Figures 1–4.

## Results

### Sucrose Alone Does Not Overcome the Light Requirement for Root Growth

To determine the effect of sucrose on root development, we used an adapted D-Root system (Silva-Navas et al., 2015) to grow Arabidopsis seedlings in a square Petri dish with their shoots exposed to light and roots in darkness (Miotto et al., 2019), mimicking the natural root growth environment. We, therefore, compared the effect of exogenous sucrose in fully illuminated seedlings (L condition), illuminated shoots and dark roots (LD condition), and seedlings grown in complete darkness (D condition). As shown in Figure 1A, 7 days after germination (DAG), seedlings grown without sucrose in L or LD showed similar primary root lengths, whereas D seedlings showed shorter main roots when compared with the other two conditions. The addition of sucrose to the culture media increased the primary root length in all conditions. Even though sucrose could induce primary root growth in D seedlings, it was not enough to reach the light effect observed in L and LD seedlings. These results indicate that, in addition to photosynthetic sugar, the light-activated signaling seems to be essential for full induction of primary root growth. To evaluate if a short light exposure would be sufficient to activate sugar-dependent root growth, we checked the effect of LD and dark-light transitions in the primary root growth. On the one hand, (1) 4 DAG seedlings grown in LD with or without sucrose were shifted to D for additional 6 days. On the other hand, (2) 4 DAG seedlings were grown in D with or without sucrose and then their shoots were exposed to 8-h light and then returned to darkness for additional 6 days. In both cases, seedlings kept in darkness developed short roots that did not activate growth by sucrose supplementation (Figure 1B). Therefore, these experiments suggest that shoot illumination is essential to promote root growth. Moreover, a short (8 h) light treatment is not enough to fully activate root growth if plants are transferred back to darkness. Taken together, these results indicate that, whereas sucrose can induce root development, exogenous sugar supply is not sufficient to overcome the continuous shoot illumination requirement as a root growth signal.

It has been shown that darkness reduces meristem size and inhibits cell proliferation (Vernoux et al., 2012). Accordingly, we tested the meristematic activity in our system. According to the *CYCB1;1:GUS* reporter (Ferreira et al., 1994), seedlings grown in complete darkness showed reduced mitotic activity and smaller root meristem than LD seedlings (Supplementary Figures 1A,B), suggesting that the short root observed in D seedlings reflects a reduction in meristem size and cell proliferation.

### Light Intensity Regulates Arabidopsis Root Growth

It has been reported that changes in light intensity lead to alterations in plant phenotype as well as in hormone homeostasis (Novák et al., 2015; Kumari et al., 2019). To gain insight into how root growth is controlled by shoot illumination, we analyzed seedlings grown in L and LD over time. WT seedlings showed an increase in primary root length in response to light intensity, reaching a plateau at 60 μmol m^–2^ s^–1^ (Figure 2A), which was sustained toward the higher intensities (80 and 105 μmol m^–2^ s^–1^). This result was consistent in 6-, 10-, and 14-day-old seedlings. In agreement with previous reports (Silva-Navas et al., 2015), the main roots displayed a slightly increased growth in LD conditions compared to L. No LR development was observed in all the evaluated time points for the low light intensity condition (40 μmol m^–2^ s^–1^) (Figure 2B). LR density was increased in higher light intensities, but no significant differences were observed among 60, 80, and 105 μmol m^–2^ s^–1^. These results clearly indicate a light dose-dependent behavior for root growth parameters indicated by a threshold between 40 and 60 μmol m^–2^ s^–1^.

The shorter primary roots observed at 40 μmol m^–2^ s^–1^ as well as the absence of LR may be related to the lower photosynthetic output from the shoots. As sucrose can stimulate primary root growth in dark-grown seedlings (Kircher and Schopfer, 2012), we wondered whether sucrose supplementation could rescue the poor root development observed at low light intensities. To test this, we grew seedlings supplemented with sucrose in low (40 μmol m^–2^ s^–1^) or medium (80 μmol m^–2^ s^–1^) light intensities. The addition of sucrose to the medium was able to increase primary root growth and LR density of the 40 μmol m^–2^ s^–1^ seedlings to match the levels of 80 μmol m^–2^ s^–1^ grown seedlings (Figures 2C,D). Nevertheless, the root growth differences between low and medium light intensities were still significant within the same growth conditions. These results suggest that root development is dependent both on shoot-derived photosynthates and on light activation of root growth. This observation indicates that signaling components need to be activated by shoot illumination to fully activate root development. This light dose-dependent response will be crucial to evaluate the downstream signaling components involved in the quantitative modulation of root growth parameters.

### Mutants of Root Light-Responsive Genes Display Changes in Root Development Under Different Light Intensities

Aiming to establish a set of genes involved in the early photomorphogenic root development of dark-grown roots, we further evaluated candidate genes identified in a previously published LD/D root transcriptomic study by Miotto et al. (2019). We selected genes that have already been described as involved in light responses, root development, and signal transduction. From the differentially expressed genes selected, genes such as *AIAMT1*, *PILS4*, *PIN5*, *PIN6*, and *YUC8* were selected to assess the effect of shoot illumination on root auxin homeostasis, whereas *ROPGEF4*, *LBD29*, *NRT2.1*, and *Shaven2* were selected for their putative roles on root development. Genes involved in response to stimulus and signal transduction such as *BTB-POZ*, *MAKR6*, *NPH3*, *ROP9*, *RIC1*, *KAI2*, *NPY4*, *PP2A-B*, and *Vid-27* were selected to investigate the downstream light signaling components. Most of the selected genes are upregulated in roots grown in LD, with the exception of *Vid-27* and *PILS4*, which are downregulated in the same conditions (Supplementary Figure 3).

To investigate whether these selected genes are involved in quantitative root growth responses to shoot illumination, we measured primary root length and LR density of T-DNA insertion lines grown in low (40 μmol m^–2^ s^–1^) and medium light intensity (80 μmol m^–2^ s^–1^). We found no significant differences in primary root growth for most of the T-DNA lines when compared to WT in both light intensities (Figure 3A). However, *lbd29* and *yuc3* mutants showed shorter roots than WT at 7 days in 80 μmol m^–2^ s^–1^, whereas *nrt2.1-1* and *shy2-2* showed shorter roots in both light intensities. The *kai2* mutant had shorter primary roots only at 40 μmol m^–2^ s^–1^. The differences observed at 7 days to *lbd29*, *nrt2.1-1*, and *kai2* mutants were no longer observed in later time points. At 10 days, only *shy2-2* presented shorter roots than WT. The *yuc3*, *shy2-2*, *pin5*, and *pils4* mutants showed shorter roots than WT in 14 days at 80 μmol m^–2^ s^–1^ only. We also observed that most genotypes displayed proportionally longer primary roots in medium light intensity. Conversely, *pin5-5*, *vid-27*, and *yuc3* mutants did not respond to higher light intensities in all evaluated time points, suggesting that these mutations impact the quantitative response of primary roots to shoot illumination.

The LR development was shown to be modulated by differences in light intensity (Kumari et al., 2019). In agreement with previous reports, LR density was strikingly affected by the light intensity to which the shoots were exposed (Figure 3B). Overall, low light strongly impaired LR development for most genotypes. At 14 days, *gef4*, *nrt2-1*, *ric-1*, *makr6*, *pp2a-b*, *roc1*, *yuc8*, *yuc9*, and *aimt1* all produced more LR than WT grown at 80 μmol m^–2^ s^–1^, suggesting that these genes act as the repressors of LR formation in response to light intensity. Only *vid-27* and *pils4* lines showed higher LR density at 40 μmol m^–2^ s^–1^ than at 80 μmol m^–2^ s^–1^. The *lbd29*, *scar1*, *shaven2*, *btb-poz*, *kai2*, and *pin5* lines did not produce LRs at low light intensities even at 14 days. The auxin-related mutants *yuc3* and *shy2-2* presented no LR development in any light condition, possibly due to changes in auxin homeostasis in these lines. The *gef4* and *nrt2.1-1* lines showed an increase in LR density in both time points at 80 μmol m^–2^ s^–1^. Our results indicate that shoot illumination strongly controls the root growth responses in Arabidopsis seedlings and that some phenotypes are highly dependent on the light intensity.

### Indole-3-Acetic Acid Effect on Primary Root Growth Is Light-Intensity Independent

Based on the phenotype showed by the auxin-related T-DNA insertion lines, we decided to check the effect of the auxin transport inhibitor NPA and IAA in the main root length combined with different light intensities. Both NPA and IAA caused a reduction in root length when supplied to the medium (Figure 4). The main root growth was strongly repressed by IAA regardless of the light treatment as opposed to what was observed in NPA treatments where higher light intensities progressively attenuated NPA negative effects on root growth. This suggests that the shoot light stimulus proportionally increases auxin polar transport to induce primary root growth not necessarily increasing root IAA concentration.

## Discussion

The effects triggered by light in plant morphogenesis were mostly studied in shoots and hypocotyls. Nevertheless, a substantial part of the plant grows protected from light, and it can adjust its development to many different factors perceived above the ground (Li et al., 2021). In this study, employing a modified D-Root system (Silva-Navas et al., 2015), we addressed how light perception in the shoot influences root development in a quantitative manner. Using our modified D-Root system, we have shown that exogenous sucrose induces root growth when roots are kept in darkness and that light intensity proportionally enhances the sugar effect. When roots are directly exposed to light, as normally happens in Petri dishes, they show a reduction in primary root length when compared to roots grown in the LD condition (Silva-Navas et al., 2015). Our experimental setup differs from previous reports where WT plants were not affected on primary root length by light intensity, but roots were exposed to direct illumination in a sucrose-enriched medium (Kumari et al., 2019). The same study reported that the *phyA-211* mutant had shorter primary roots and lower LR formation under lower light intensities. It has been described that both shoot- and root-expressed phytochromes can trigger downstream signaling responses in illuminated roots (Salisbury et al., 2007). We have observed a threshold-like behavior of root growth in response to increasing shoot illumination. Root growth can be further stimulated by exogenous sucrose, which suggests demand for photosynthetic carbon to enhance both primary root growth and LR emergence.

It has been reported that higher intensities (280 μmol m^–2^ s^–1^) have an inhibitory effect on root elongation (Silva-Navas et al., 2015), reinforcing the idea that roots sense direct illumination as a stress (Yokawa et al., 2011, 2014). Leaves upload photosynthates such as sucrose into the vasculature that sustain the growth of non-photosynthetic organs. Photosynthesis controls the metabolic activation of root meristems *via* sugar signaling (Xiong et al., 2013) and glucose acts in concert with auxin to sustain the main root growth (Mishra et al., 2009). In our experiments, dark-grown seedlings could activate main root growth by externally adding sucrose as in previous reports (Kircher and Schopfer, 2012). Interestingly, light and sucrose synergistically promoted the emergence of LRs whereas the light intensity proportionally enhanced the sugar effect. Additionally, seedlings growing in the light, once transferred to total darkness, ceased main root growth regardless of the presence of sucrose. These findings indicate that light intensity and main root development are dependent on photoreceptor signaling for sucrose to be effective. Additionally, our data indicate that sugar and light act synergistically, and a constant light signaling input is necessary for sucrose assimilation by the roots.

Few of the genes that were evaluated in this study such as *VID-27*, *PILS4*, *PIN5*, and *YUC8* showed higher expression in dark-grown etiolated roots. In addition, most of the evaluated mutants, except for *nph3* and *yuc8*, showed shorter roots than WT when shoots were exposed to light, suggesting a positive role in primary root growth. Future studies will be necessary to address accurately the required function of these genes for primary root development. Control of root structure and development is regulated by phytohormone synthesis and distribution. Remarkably, in *yuc3* and *pin5* mutants, the primary root development was not responsive to increasing light intensities. This suggests that these genes may act modulating the quantitative light response of dark roots. Nevertheless, it shows that both light- and auxin-regulated pathways interact to regulate dark-grown root development. From all the candidate genes analyzed in this study, it was observed that the vacuolar import/degradation Vid27-related protein was the only mutant that presented an increase in LR density in the lower light intensity. In addition, its primary root development was not differentially affected by 40 and 80 μmol m^–2^ s^–1^ light intensities. Moreover, the transcriptome analysis shows that *vid27* is repressed in roots by shoot illumination. These results suggest that this gene may work as a repressor of LR development in seedlings exposed to light.

In this study, we have shown that root growth responses are drastically affected by shoot illumination. The light intensity affects primary and LR development in Arabidopsis in a dose-dependent manner. Our results indicate that some mutants only display root growth-related phenotypes at certain light intensities and that light perception modulates growth responses more strongly through photoreceptor activation than on photosynthate availability. These results question the best practices for Arabidopsis growth *in vitro* and reinforce the benefits of a D-Root system to avoid the adverse effects of direct illumination impinge on root physiology (Xu et al., 2013; Yokawa et al., 2014; Novák et al., 2015; Silva-Navas et al., 2015; Qu et al., 2017). Elucidating the possible long-distance signaling from illuminated shoot to trigger early root photomorphogenesis will need further experiments, but our findings suggest that sucrose and auxin act in a positive way to coordinate root development in response to light. Further investigation of the specific role of candidate genes in this response may help us to better understand how plants adapt root development in response to external stimuli.

## Data Availability Statement

The additional data generated in the study are included in the article/Supplementary Material, further inquiries can be directed to the corresponding author.

## Author Contributions

YM, CC, RO, JK-V, and FM conceived the experiments. YM, CC, and FM performed the experiments and analyzed the results. YM and FM wrote the manuscript. All authors contributed to the revision of the manuscript and read and approved the submitted version.

## Conflict of Interest

The authors declare that the research was conducted in the absence of any commercial or financial relationships that could be construed as a potential conflict of interest.

## Publisher’s Note

All claims expressed in this article are solely those of the authors and do not necessarily represent those of their affiliated organizations, or those of the publisher, the editors and the reviewers. Any product that may be evaluated in this article, or claim that may be made by its manufacturer, is not guaranteed or endorsed by the publisher.

## Footnotes

1
http://arabidopsis.info/

2
http://signal.salk.edu/index.html

## Supplementary Material

Supplementary Figure 1 Relative light intensity experienced by roots in the modified D-Root growth system used in the experiments (LD condition). The graph shows the light intensity in relation to the depth of the black paper cover. The top intensity of 80 μmol m^–2^ s^–1^ was set as 100%, and the relative intensities were measured inside the Petri dishes to obtain the relative values.

Supplementary Figure 2 **(A)** Root apical meristem and **(B)** *CYCB1;1:GUS* expression patterns of 4-day-old D and LD grown seedlings. Asterisks denote significant differences against the control by the Student’s *t*-test (^∗∗^*p* < 0.001).

Supplementary Figure 3 Candidate genes expression in root tissues. Gene expression was normalized using an internal control (AT3G18860 and AT2G42500; Miotto et al., 2019) for each reaction. The expression levels of the 4 DAG D condition were set as 1.
