# Supplementary figures and images for: Effects of Light Intensity on Root Development in a D-Root Growth System
Source: Front Plant Sci. 2021 Dec 15;12:778382. doi: 10.3389/fpls.2021.778382 (PMC8715079; doi:10.3389/fpls.2021.778382)

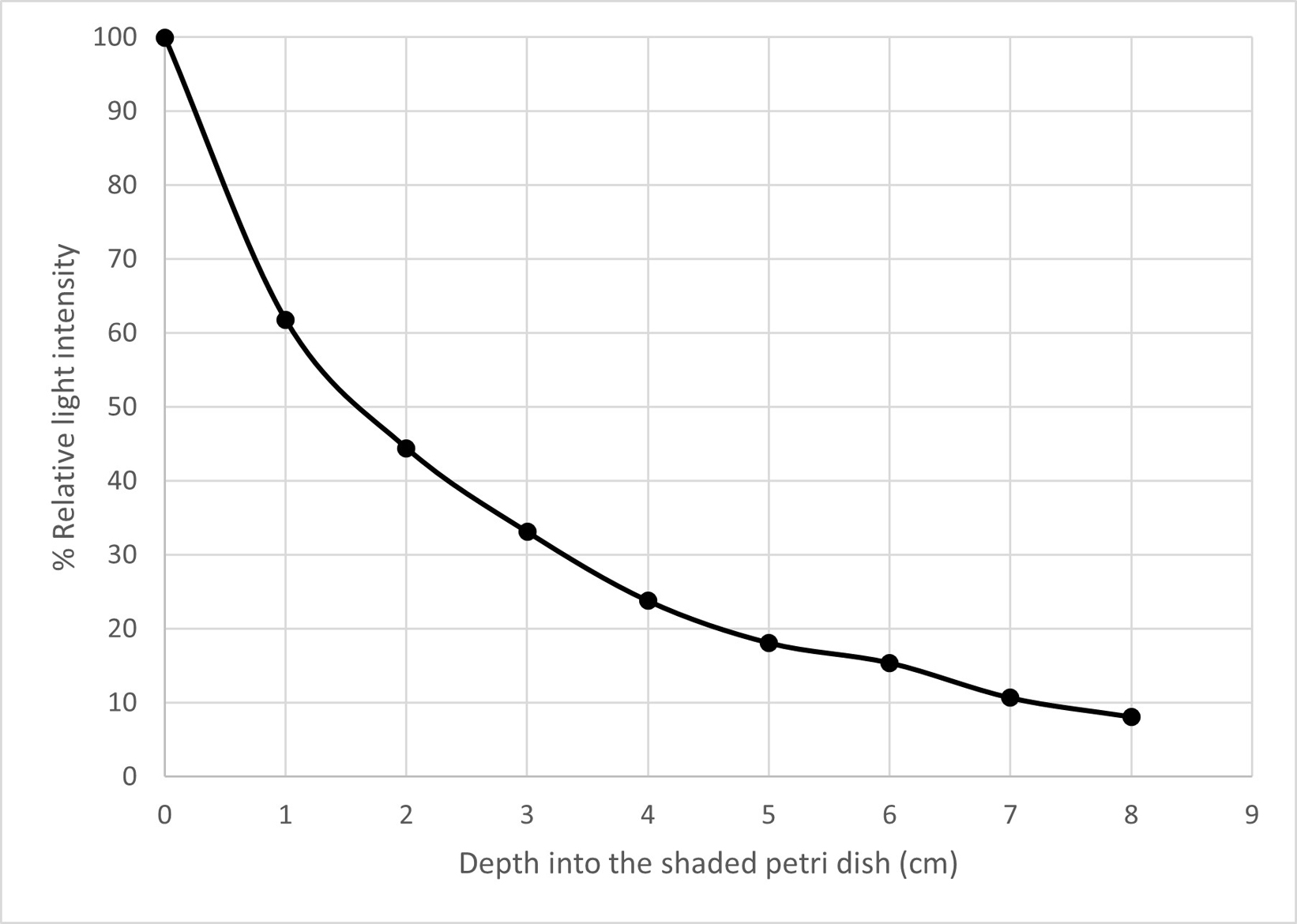

Supplement: Supplementary Figure 1 — Relative light intensity experienced by roots in the modified D-Root growth system used in the experiments (LD condition). The graph shows the light intensity in relation to the depth of the black paper cover. The top intensity of 80 μmol m–2 s–1 was set as 100%, and the relative intensities were measured inside the Petri dishes to obtain the relative values. [file Image_1.JPEG]

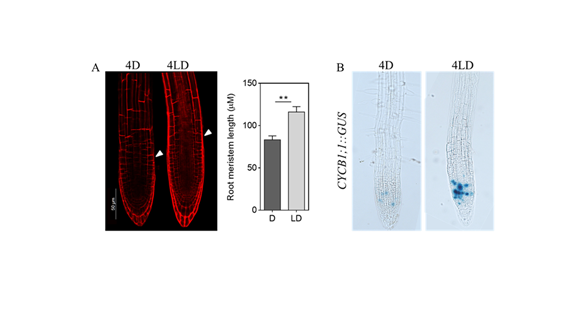

Supplement: Supplementary Figure 2 — (A) Root apical meristem and (B) CYCB1;1:GUS expression patterns of 4-day-old D and LD grown seedlings. Asterisks denote significant differences against the control by the Student’s t-test (∗∗p < 0.001). [file Image_2.TIF]

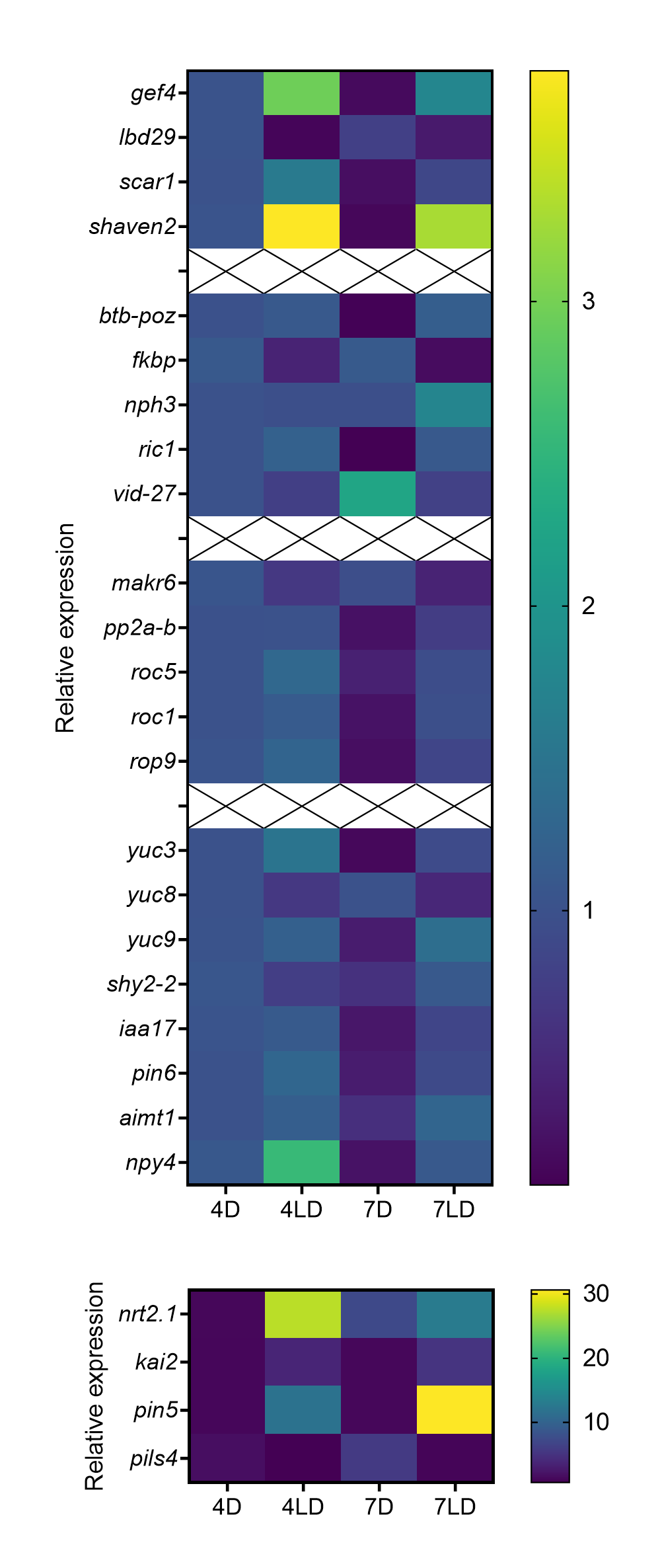

Supplement: Supplementary Figure 3 — Candidate genes expression in root tissues. Gene expression was normalized using an internal control (AT3G18860 and AT2G42500; Miotto et al., 2019) for each reaction. The expression levels of the 4 DAG D condition were set as 1. [file Image_3.TIF]
